# Birth preparedness and complications readiness among women in a cosmopolitan city in western Nigeria: a mixed-methods cross-sectional study

**DOI:** 10.1186/s41043-026-01420-9

**Published:** 2026-08-17

**Authors:** Ifeoma Peace Okafor, Mobolanle Balogun, Adekemi Sekoni, Duro Dolapo, Claudia Hawkins, Bosede Bukola Afolabi

**Affiliations:** 1 Department of Community Health & Primary Care, College of Medicine, University of Lagos, Lagos, Nigeria; 2 Department of Community Medicine, Nile University of Nigeria, Abuja, Nigeria; 3 Institute for Global Health- Centre for Global Communicable and Emerging Infectious Diseases, Northwestern University, Chicago, IL USA; 4 Department of Medicine, Northwestern University Feinberg School of Medicine, Chicago, IL USA; 5 Department of Obstetrics & Gynaecology, College of Medicine, University of Lagos, Lagos, Nigeria

## Abstract

**Background:**

Low- and middle-income countries bear a significant burden of the global maternal and neonatal mortality. Birth preparedness and complications readiness (BP/CR) reduces the risk of preventable deaths.

**Objective:**

To assess BP/CR and predictors among recently delivered women in urban Lagos, southwest Nigeria.

**Methods:**

This was a mixed-methods cross-sectional, community-based study involving urban women who delivered within two years prior to study. Recruitment was by multistage sampling method. Quantitative data were collected from 790 women using a validated questionnaire. Respondents’ level of BP/CR was measured using key components of BP/CR. Summary and inferential statistics were done. Predictors of BP/CR were determined by multivariable regression analysis. Focus group discussions (FGDs) were used to collect exploratory qualitative data using a semi-structured interview guide and were analyzed thematically.

**Results:**

Mean age of 790 respondents was 31.1 years (SD 1.8). About 78% utilized formal antenatal care services and institutional delivery (74.6%) in their last pregnancy. Reported counselling on BP/CR was inadequate. Only 26.6% (95% CI 23.5 to 29.7) met three out of the four key BP/CR components hence ‘well prepared’ − 94.8% saved money towards the baby’s birth, 33.5% identified mode of transport ahead of labour or emergency, 29% identified health facility (HF) or skilled birth attendant (SBA) for delivery, and 9.4% identified blood donor for emergency. The predictors of being ‘well prepared’ were, older age ≥ 30 years (AOR: 1.80; 95% CI: 1.25, 2.59), Yoruba ethnicity (AOR: 1.49; 95% CI: 1.04, 2.11), monthly income/allowance of less than the minimum wage (AOR: 1.73; 95% CI: 1.23, 2.42), lower parity < 5 (AOR: 2.96; 95% CI: 1.28, 6.86), final decision on BPCR by mother or relatives (AOR: 2.00; 95% CI: 1.00, 4.00), recent delivery in the last six months prior to study (AOR: 1.64; 95% CI: 1.11, 2.43), previous delivery in HF (AOR: 1.58; 95% CI: 1.05, 2.36), and receiving counselling on facility delivery/SBA (AOR: 1.64; 95% CI: 1.15, 2.34). FGDs revealed that traditional obligations, bureaucratic delays, inadequate finance, and poor health worker attitudes, constitute challenges to adequate BP/CR practice.

**Conclusion:**

The urban women were not prepared for birth and possible complications. There is urgent need for integrated interventions that address individual, family, community, and health system barriers to BP/CR practice to reduce maternal and newborn deaths and achieve sustainable development goals (SDG) 3.

## Introduction

 Maternal and newborn mortality has remained persistently high in low and middle income countries (LMICs), accounting for over 90% of the burden of 223 per 100,000 live births globally [1]. Major targets of the sustainable development goals (SDG) 3 include- by 2030: reduce maternal mortality ratio (MMR) to less than 70 per 100,000 live births, end preventable newborn and child deaths with countries aiming to reduce neonatal mortality to as low as 12 per 1000 live births, and under five (U5) mortality to as low as 25 per 1000 live births. The latest report so far shows a global decline in MMR by only 14% and U5 mortality rate by only 16% [2]. 

“Nigeria has one of the highest burdens of maternal mortality globally” [3] The MMR in Nigeria is estimated to be 1047/100,000 live births [4] and for every maternal death, 20 or more other mothers suffer serious morbidities [5]. Neonatal mortality rate is 41/1000 live births and only 46% of deliveries are performed by a skilled provider [6]. The stillbirth rate at 20 weeks or longer (53.6/1000 births) is the third highest in Sub-Saharan Africa [7] and is a strong indicator of the quality of care during pregnancy and childbirth [8]. 

Most newborn and maternal deaths are preventable with adequate emergency obstetric and newborn interventions. Birth Preparedness and Complication Readiness (BP/CR) is a process of preparing or planning for birth and anticipating what to do in the event of obstetric complications [9]. In terms of BP/CR conceptual framework, the key components cover the knowledge of danger signs, advancepreparation for delivery by saving money, identifying a skilled birth attendant (SBA) and the location of the nearest health facility with emergency obstetric care (EmOC), arranging for transportation, and potential blood donor (or donating blood). Others are identifying someone to accompany the woman and temporary family care in case of emergencies [9]. These are all targeted at safe delivery even when faced with emergency to achieve best pregnancy outcomes. The framework emphasizes the vital roles of shared responsibility among key stakeholders- the pregnant women and their families, the community members, healthcare facilities/providers and policy makers. Pregnant women and their partners can prepare by learning to recognize obstetric and newborn danger signs that may indicate life-threatening complications, identifying a skilled birth attendant/health facility, saving money, and arranging for transportation. Families and communities can prepare by planning for money, transport, or a blood donor to facilitate reaching and receiving care in case of an obstetrical emergency. Health facilities can prepare by having the required equipment, supplies, and support systems readily available. Clinical personnel can prepare by acquiring the necessary knowledge and skills needed to attend normal childbirth and manage complications. Policymakers can prepare by instituting evidence-based healthcare policies and assuring adequate funding for maternal and newborn healthcare services.

This concept of ‘preparation in advance’ helps to avoid delays in deciding, reaching, and receiving appropriate care, thus preventing poor maternal and neonatal health outcomes [10]. It enhances access to skilled care, facilitates quick response to complications, empowers families to be involved in care and make informed decisions, and encourages communities to be proactive in preparing for safe childbirth [9]. BP/CR plans are included in the WHO integrated management of pregnancy and childbirth (IMPAC) [11] and a systematic review of randomized controlled trials showed that BP/CR strategies can indeed be effective in reducing neonatal and maternal death risk [12]. 

In Nigeria, birth preparedness was launched in 2005 by the Federal Ministry of Health with limited results [13]. Studies from Nigeria have shown a positive relationship between BP/CR and maternal and neonatal pregnancy outcomes [14, 15]. However, there is a wide variation in the proportion of women being birth prepared and complications ready. Some recent studies have reported low proportions (below 50%) and predictors such as having ANC, higher educational level, receiving counselling on BP/CR and history of still birth among others [16–23]. 

The study was conducted to assess the prevalence of being birth prepared and complication ready, and predictors among recently delivered women in Lagos, southwest Nigeria. Lagos State is largely urban and boasts of diverse demographic representation of different tribes, religions, occupations and economic backgrounds with a sizeable proportion of ‘urban poor’. The health system is quite robust when compared with many states in the country but there are also alternative healthcare providers practicing in the state. Similar studies done in western Nigeria have largely been facility-based, or rural and involved fewer respondents.

Findings will elucidate prevailing factors regarding BP/CR and further guide appropriate interventions to facilitate a rapid and sustained reduction in preventable maternal and neonatal deaths.

## Methodology

### Study setting

The study was carried out in Kosofe and Ifako-Ijaiye Local Government Areas (LGAs), Lagos State, Nigeria. These urban LGAs had the highest maternal mortality in the state from available data at the time of commencing the study (378 and 561per 100,000 live births respectively) [24]. Ifako-Ijaiye has 14 political wards and Kosofe has 21. From the 2006 census and Lagos State growth rate of 3.2%, the projected population of Ifako Ijaiye LGA is 1,019,902 and Kosofe LGA is 1,280,646 [25]. There are many health facilities, both public and private in the study areas, as well as alternative maternity care providers, such as traditional birth attendants (TBAs).

### Study population and eligibility criteria

The study population included mothers in Kosofe and Ifako-Ijaiye. Inclusion criteria were - delivered a baby in the two years prior to interview and being a permanent resident. The exclusion criterion was inability to respond to questions appropriately due to ill health or cognitive challenges.

### Study design and sample size

This was a cross-sectional study employing mixed data collection methods. The sample size was calculated by applying the Cochran formula [26] using the proportion of women who were ‘well prepared’ (62%) [27], level of significance of 95% (1.96), a margin of error of 5% and design effect of 2. The calculated figure (724) was increased by 10% (796)to cover for non responses and rounded up to 800.

### Sampling method

Multistage sampling was used in selecting respondents. In the first stage, two wards were selected from each LGA from the list of wards using simple random sampling (balloting). Respondents were equally allocated to the four selected wards, approximately 198 per ward. Next was the second stage where 10 streets were selected from the list of streets in each ward using simple random sampling. The third stage was selection of houses using systematic sampling based on a sampling interval, k which was calculated based on the number of respondents required and the no of houses per street. More houses were selected from longer streets. The starting point was selected by simple random sampling (balloting) between first to kth house. Houses consisted of several households. In the fourth stage, households were selected from houses by simple random sampling (balloting), one household per house. Finally, the fifth stage (selection of respondents) where research assistants (RAs) identified women who met the inclusion criteria for interview. Only one woman was interviewed in each selected household. Data collection was done over two months, July and August 2019.

**Data collection methods and tools**: Quantitative and qualitative data collection methods were employed in this study. Quantitative data were collected by six trained RAs using structured, pretested, interviewer-administered questionnaires adapted from a validated tool from JHPIEGO [9]. The questionnaire was organized into sections to elicit relevant information from the respondents. Sociodemographic characteristics covered include age, marital status, ethnicity and highest educational level. For their economic characteristics, respondents were asked whether they were employed with a ‘Yes’ or ‘No’ response. Respondents reported their monthly income if employed or estimated their monthly allowance/support if unemployed. Other data collected were obstetric characteristics such as parity, history of child death and time of last delivery; the place of ANC and delivery services; counselling on BP/CR received; and their practice of basic components of BP/CR in the index pregnancy namely: save money, identify health facility/SBA, identify/arrange for transport, and identify blood donor/donate blood.

The RAs all had good communication skills, possessed university degree qualifications and were fluent in English, Yoruba and Pidgin English, which are the commonly spoken languages in the study areas.

The qualitative aspect was designed to obtain more insight and context and complements the quantitative study. One focus group discussion (FGD) comprising 8 women per group was done in a neutral venue in each LGA using a pretested, semi-structured interview guide developed by the researchers to further explore the determinants of women’s practices regarding BP/CR in the community. The FGDs were conducted by the lead researcher assisted by three RAs and discussants were purposively recruited with the assistance of women community leaders following the same inclusion criteria for the quantitative study. Diverse perspectives were captured, and saturation was determined when no new ideas or themes were added to the data. Discussions were audio-taped in addition to field notes and transcribed verbatim.

### Data management and analysis

Quantitative data were analyzed with IBM-SPSS version 25 software. Categorical data were summarized using proportions, continuous data with mean and standard deviation (SD) and discrete data with median and interquartile range. Chi square (*χ*²) tests were used to assess associations between categorical explanatory variables and BP/CR status.

Multiple logistic regression was used to identify the predictors of ‘being well prepared’ for childbirth and possible complications. A p-value cut-off of 0.1 in bivariate analyses was used to include variables into the regression model [28]. The level of significance was 5% (*p* ≤ 0.05).

‘Framework’ analysis method was applied for the analysis of qualitative data [29]. 

A combination of inductive and deductive approaches was used. Attribute codes were assigned and categorized, and themes were developed both from the research questions and the narratives of the discussants. Emerging patterns were noted and refined with subsequent rounds of reading and analysis to gain more insight into factors influencing BP/CR. A final report was written based on this. Relevant quotes were highlighted and selected quotes were captured under the thematic contents and interpreted.

**Measurements**: BP/CR was scored and categorized using only the basic components of BP/CR [9] and adaptations from previous similar studies [16, 17]. Each basic component mentioned as practiced attracted one point: save money, identify health facility/SBA, identify/arrange for transport, and identify blood donor/donate blood. Minimum and maximum scores were zero (0) and four (4) representing 0% and 100% respectively. Result was dichotomized: respondents who practiced at least 3 basic components of BP/CR were considered ‘well prepared’ and less than 3, ‘not well prepared’ for birth and obstetric complications.

## Results

### Quantitative

#### Sociodemographic and obstetric characteristics

Seven hundred and ninety questionnaires were adequately filled and eventually analyzed. Mean age was 31.1 years, standard deviation (SD) 1.8. The majority were married (92.5%) and most completed secondary school (60.5%). Most women were employed (80.5%); 46.5% earned below the minimum wage monthly (about 90 USD at the time of study).

Most women had between one to three children (82.4%), had ANC in health facility (77.7%) and institutional delivery (74.6%). Decisions regarding the respondent’s pregnancy were usually taken jointly with the spouse (47.6%) [Table 1].

**Table 1:** Sociodemographic and obstetric characteristics of respondents

Sociodemographic characteristics	Frequency (*N* = 790)	Percentage (%)	Obstetric characteristics	Frequency (*N* = 790)	Percentage (%)
**Age group (years)**			**Parity**	176	22.3
< 20	13	1.6	1	249	31.5
20–29	301	38.1	2	195	24.7
30–39	412	52.2	3	112	14.2
≥ 40	64	8.1	4	58	7.3
Mean (SD)	31.1 (1.8)		≥ 5	2 (2–3)	
**Marital status**			Median (IQR)		
Married	731	92.5	**History child death**		
Not married	59	7.5	Yes	107	13.5
**Ethnicity**			No	683	86.5
Yoruba	489	61.9	**No of living children**		
Igbo	151	19.1	1	203	25.7
Others	150	19.0	2	266	33.7
**Religion**			3	182	23.0
Christianity	551	69.7	*4*	96	12.2
Islam	239	30.3	≥ 5	43	5.4
**Highest educational level**			Median (IQR)	2 (1–3)	
No formal	20	2.5	**Place of last ANC**		
Primary	124	15.7	Did not have ANC	61	7.7
Secondary	478	60.5	Only health facility	614	77.7
Post-secondary	168	21.3	Only TBA	60	7.6
**Employed**			Both HF and TBA	55	7.0
Yes	634	80.3	**Last delivery time (months)**		
No	156	19.7	< 6	163	20.6
**Monthly income/allowance (₦)**			6–11	182	23.0
< 30,000	366	46.3	12–17	191	24.2
30,000–39,000	158	20.0	18–24	254	32.2
40,000–49,000	88	11.1	Mean (SD)	12.9 (7.4)	
≥ 50,000	178	22.5	**Place of last delivery**		
Mean (SD)	35,670 (36,911)		Health facility	589	74.6
**Major decision maker for respondent’s pregnancy**			TBA	92	11.6
Respondent	219	27.7	Religious home	51	6.5
Respondent and spouse	376	47.6	Respondent’s Home	31	3.9
Spouse	70	8.9	Others	27	3.4
Mother-in-law	47	5.9			
Respondent’s mother or relatives	38	4.8			
Others	40	5.1			

TBA- Traditional birth attendant; HF- Health facility

### TBA- Traditional birth attendant; HF- Health facility

#### Counselling on the key components of BP/CR

Almost 60% received counselling on delivery in HF/Skilled birth attendant (SBA), 41% on saving money or making arrangements for funds, 15% on arrangement for transportation, and 12.2% on identifying blood donor.

**BP/CR practice**: Overall, 26.6% (95% CI: 23.5 to 29.7) met three out of the four key BP/CR components and thus were ‘well prepared’ while 73.4% (95% CI: 70.3 to 76.5) did not, thus were ‘not well prepared’. Almost 95% saved money or made arrangements for funds, 33.5% identified mode of transport, 29% identified HF/SBA, and 9.4% identified blood donor (Table 2).

**Table 2:** Counselling and Practice of BP/CR among respondents

Variable	Frequency (*n* = 790)	Percentage	95% CI
^#^What counselling was received on			
Delivery in facility/SBA	471	59.6	
Saving money	324	41.0	
Arrangements for transport	118	14.9	
Arrangements for blood donor	96	12.2	
^**#**^ **Practice of BP/CR**			
Made any arrangements for childbirth	743	94.1	
Saved money	705	94.8	
Identified mode of transport	250	33.5	
Identified HF/SBA	215	29.1	
Identified blood donor/donated blood	73	9.4	
*Overall level of BPCR			
Well prepared	210	26.6	23.5–29.7
Not well prepared	580	73.4	70.3–76.5

^#^Multiple responses *At least three out of four basic components = Well prepared; CI- Confidence Interval

#### Factors associated with BP/CR

In bivariate analyses, employment status (χ^2^ = 5.443, *p* = 0.020), monthly income (χ^2^ = 11.187, *p* = 0.001), parity (χ^2^ = 6.756, *p* = 0.009), time of last delivery (χ^2^ = 6.359, *p* = 0.012), previous HF delivery (χ^2^ = 5.283, *p* = 0.022), major decision maker on respondent’s pregnancy (χ^2^ = 6.742, *p* = 0.009), and receiving counselling on delivery in HF/SBA (χ^2^ = 12.801, *p* < 0.001) were all associated with BP/CR [Table 3].

**Predictors of BP/CR**: In multiple logistic regression, after adjusting for effects of confounders (including interaction effects of age and level of education), predictors of being well prepared for childbirth and obstetric complications were: older age ≥ 30 years (Adjusted OR (AOR): 1.82; 95% CI: 1.26, 2.61), belonging to Yoruba ethnic group (AOR: 1.49; 95% CI: 1.04, 2.11), monthly income/allowance of less than the minimum wage (AOR: 1.73; 95% CI: 1.23, 2.42), having parity < 5 (AOR: 2.96; 95% CI: 1.28, 6.86), final decision on BP/CR being made by mother or relatives (AOR: 2.00; 95% CI: 1.00, 4.00) having a baby in the last six months prior to study (AOR: 1.64; 95% CI: 1.11, 2.43), previous delivery in HF (AOR: 1.58; 95% CI: 1.05, 2.36) and being counselled on facility delivery/SBA (AOR: 1.64; 95% CI: 1.15, 2.34) [Table 3].

**Table 3:** Associated factors and predictors of BP/CR

Variable			Well prepared	Total	χ^2^	*p*-value
			*n* = 210 (26.6%)	*n* = 790		
**Age group (years)**						
< 20			2 (15.4)	13	3.363	0.339
20–29			73 (24.3)	301		
30–39			120 (29.1)	412		
≥ 40			15 (23.4)	64		
**Marital status**						
Married			194 (26.5)	731	0.090	0.923
Not married			16 (27.1)	59		
**Highest educational level**						
No formal			7 (35.0)	20	1.269	0.737
Primary			30 (24.2)	124		
Secondary			126 (26.4)	478		
Post-secondary			47 (28.0)	168		
**Ethnicity**						
Yoruba			143 (29.2)	489	4.667	0.097
Igbo			34 (22.5)	151		
Others			33 (22.0)	150		
**Employed**						
Yes			157 (24.8)	634	5.443	0.020*
No			53 (34.0)	156		
**Monthly income (₦)**						
< 30,000			118 (32.2)	366	11.187	0.001*
≥ 30,000			92 (21.7)	424		
**Parity**						
< 5			203 (27.7)	732	6.756	0.009*
≥ 5			7 (12.1)	58		
**History child death**						
Yes			27 (25.3)	107	0.115	0.734
No			183 (26.8)	683		
**Last delivery time (month)**						
< 6			56 (34.4)	163	6.359	0.012*
≥ 6			154 (28.0)	627		
**Previous HF delivery**						
Yes			169 (28.7)	589	5.283	0.022*
No			41 (19.6)	201		
**Final decision is taken by mother or relatives**						
Yes			17 (44.7)	219	6.742	0.009*
No			193 (32.5)	752		
**Counselled on delivery in HF/SBA**						
Yes			147 (31.2)	471	12.801	< 0.001*
No			63 (19.7)	319		
**Counselled on arrangements for funds**						
Yes			89 (27.5)	324	0.221	0.638
No			121 (26.0)	466		
**Counselled on arrangements for transport**						
Yes			28 (23.7)	118	0.579	0.477
No			182 (27.1)	672		
**Counselled on arrangements for blood donor**						
Yes			27 (28.1)	96	0.133	0.715
No			183 (26.4)	694		

**Simple and multiple logistic regression of adequate BP/CR on the associated factors**						
**Predictor variable**	**Simple regression**			**Multiple regression**		
	**COR**	**95% CI**	**p-value**	**AOR**	**95% CI**	**p-value**
**Age (year)**						
< 30	1			1		
≥ 30	1.26	0.91–1.75	0.164	1.80	1.25–2.59	0.001*
**Yoruba ethnicity**						
No	1			1		
Yes	1.44	1.03–2.02	0.031*	1.49	1.04–2.11	0.028*
**Post-secondary education**						
No	1			1		
Yes	1.09	0.75–1.70	0.645	0.93	0.61–1.40	0.714
**Employed**						
Yes	1			1		
No	1.56	1.07–2.28	0.020*	1.48	0.99–2.22	0.056
**Monthly income/allowance**						
≥₦30,000	1			1		
<₦30,000	1.72	1.25–2.36	< 0.001*	1.73	1.23–2.42	0.002*
**Parity**						
≥ 5	1			1		
< 5	2.80	1.25–6.26	0.012*	2.96	1.28–6.86	0.011*
**Final decision by woman or relatives**						
No	1			1		
Yes	2.35	1.21–4.54	0.011*	2.00	1.00–4.00	0.049*
**Last delivery time in months**						
≥ 6	1			1		
< 6	1.61	1.11–2.33	0.012*	1.64	1.11–2.43	0.013*
**Previous health facility delivery**						
No	1			1		
Yes	1.57	1.07–2.31	0.022*	1.58	1.05–2.36	0.028*
**Counselled on facility delivery/SBA**						
No	1			1		
Yes	1.84	1.32–2.58	< 0.001*	1.64	1.15–2.34	0.006*

* Statistically significant; SBA=Skilled birth attendant; COR = Crude Odds Ratio; AOR= Adjusted Odds Ratio; CI-Confidence Interval

### Qualitative

The demographic characteristics of the focus group discussants are presented in Table 4.

**Table 4:** Socio demographic characteristics of discussants

Ifako-Ijaiye (IFK)		
Code	Age (years)	Educational level
P1	36	Secondary
P2	23	Secondary
P3	35	Post secondary
P4	40	Secondary
P5	25	Primary
P6	43	Post secondary
P7	30	Secondary
P8	22	Secondary
**Kosofe (KSF)**		
P1	28	Post secondary
P2	39	Secondary
P3	44	Secondary
P4	23	Primary
P5	31	Secondary
P6	35	Post Secondary
P7	25	Secondary
P8	40	Post secondary

Ifako-Ijaiye (IFK), Kosofe (KSF)

### Understanding of the concept of birth preparedness and knowledge of key components

Many discussants understood BP/CR, but few cited key components like saving money or identifying a HF/SBA. Emergency preparedness was rarely mentioned, with most equating BP/CR to buying baby items. Most women said preparation typically starts at 3 months of pregnancy.

*“It (BP/CR) is the preparation we do before giving birth.”* (P3, IFK, 35 years).

*“We buy baby things.”* (P3 KSF, 44 years).

### Importance of birth preparedness and complications readiness

Based on the shallow context of BP/CR presented initially by the discussants, further probing emphasizing on the emergency preparedness was done. Most discussants opined that indeed, BP/CR is for the safety of the child and mother.

*“The important thing that I know is to save life. Without life*,* all is waste….”* (P8, KSF, 40 years).

Some women with complications divert to TBAs for local concoctions instead of following medical advice. Even when advised having a caesarean section (C/S) or to donate blood for low levels, they reject it, believing that TBAs have special concoctions and powers to avoid surgery.

*“Maybe the doctor did test*,* and you’ve been told to come back for a short appointment because of the problem you have*,* but there are some pregnant women that would rather go to ‘Ile Alagbo’ (TBA). All this ‘agbo’ (local concoctions) stuff*,* they will rather add more complication to what is on ground.”* (P8, IFK, 22 years).

*“Our pregnant women think that Ile Alagbo (TBA) is preferred for delivery if the baby is coming with leg*,* or with hand because the health workers will tell them that they will have C/S but if it is TBA*,* they will do it traditionally to make the baby come out quickly.”* (P1, KSF, 28 years).

### BP/CR practices in the community

Discussants described birth preparation mainly as purchasing baby items and ANC registration. Some planned for HF delivery, others for TBA or home birth. Emergency preparedness was rarely mentioned spontaneously and only emerged after probing, reflecting an assumption of ‘smooth pregnancy and delivery’.

One discussant recounted how she met a woman who was already in labour but had no money, nor baby things and so she went to the TBA centre for cheaper cost.

*“Some will say because of money she will not go to the health centre*,* some will say because of distance she will not go to health centre she will go to Ile Alagbo (TBA).”* (P8, KSF, 40 years).

*“Some prefer to deliver at home; some will call nurse to come and take their delivery in their house.”* (P7, KSF, 25 years).

One of them expressed her ‘ideal preparation’ for birth and possible complications:

*“Number one is that the woman will buy all the things that she will need during labour*,* like all the things they use to write in the hospital for pregnant women. Number two is for someone that registered in a health facility that is far from where she lives. Transport should be ready because nobody knows the time it is*,* (ie labour) only God. So maybe the labour starts in the midnight and maybe her husband doesn’t have a car*,* or he is not around*,* and nobody there can take care of her at that time but once the person has made arrangement with a taxi driver or whoever*,* so during that period she can call the driver at that particular time.”* (P1, IFK, 36 years).

Another woman expressed that some women do not make any formal preparations but rather depend on the TBA who lives nearby.

*“Some women don’t really prepare. They use Ile Alagbo (TBA) who is living close to their house.”* (P2, IFK, 23 years).

### Challenges to birth preparedness

From the discussions, this is mainly financial (purchase of items, hospital bills, and then the ‘naming ceremony’). The naming ceremony is a culture of the Yorubas where the baby is named on the 8th day after delivery. It is usually a big, expensive celebration. To manage delivery costs, women save by using alternative maternity providers that allow instalment payments and long-term debt, options not available in health facilities. The nurses who are called for home deliveries are usually auxiliary nurses.

*“You need savings. That is why some people prefer to go to ‘Ile Alagbo’ (TBA) which is cheaper so that they can have money for the naming ceremony*,* to buy things for people during the naming. You really have to prepare yourself financially….”* (P4, IFK, 40 years).

*“Finance is number one thing. I will say it holds women back. It even drives most women to this herbal concoction stuff because they think that on getting to the hospital*,* they will be asked to drop money for this and that….”* (P8, IFK, 22 years).

*“Some of the pregnant woman say that in the hospital*,* the money is too much and so they call home nurse (auxiliary nurse) to come and take their delivery. Some after taking their delivery*,* they will not pay immediately*,* some will be paid a little amount. It still boils down to what we are talking about*,* money….”* (P5, KSF, 25 years).

*“Some prefer to deliver at home; some will call nurse to come and take their delivery in their house.”* (P7 KSF 25 years).

### **Planning for place of delivery and factors influencing or mitigating against people using health facility**

Women’s delivery plans varied: some chose private hospitals for perceived quality care, others public hospitals for affordability. Some opted for TBA centres because they were cheaper and provided herbal concoctions believed to be effective.

Barriers to public facilities included bureaucratic delays, poor staff attitudes, and perceived theft of baby/delivery items - though probing showed nurses often reallocated unused items to other women without permission. Participants also called for 24-hour PHCs closer to their communities.

In summary, inadequate finance, bureaucratic bottlenecks, long waiting time, poor attitude of HWs, no 24 h operation were mostly mentioned as barriers to health facility delivery.

*“Some women prefer private hospital because in General Hospital*,* that place is not easy at all. You will be shouting in pains*,* but they will not attend to you. They will think maybe you are just shouting for nothing.”* (P3, IFK, 35 years).

*“Some women complain about nurses*,* that they used to insult them….”* (P4, KSF, 40).

*“Our health centre closes maybe 5 or 6 o’clock and you know some women deliver in the night or in the mid night and they would have closed there. It’s not 24 hours that is why some women prefer to go elsewhere rather than health centre”.* (P2, KSF, 39 years)

*“I will say the place (General Hospital) is too far and they will abuse the women….”* (P7, KSF, 25 years).

*“Another thing*,* they normally delay them in the health centre they will not answer them on time they pick number*,* line up. They will spend the whole day. That is why some people prefer private health facilities*,* immediately in private health facilities*,* they will answer you….”* (P8, KSF, 40 years).

*“I will say distance*,* no night duty and financial aspect- these three things.”* (P3, KSF, 44 years).

### Major decision maker on BP/CR

Most times decisions are made jointly with the spouse. Further probing revealed that women generally go along with their husband’s decision because they are not financially empowered. If the woman has enough money, she is likely to decide on her own and try to convince the husband.

*“It depends on the relationship between husband and wife….”* (P7, IFK, 30 years).

*“It can come from the man or woman but mainly the man. We know he is the bread winner of the family…. mainly men but the woman is also there to support and assist sometimes……”* (P5, KSF, 31 years).

Upon integration, quantitative and qualitative findings were mostly convergent except in the area of finance as a determinant of BP/CR (Table 5).

**Table 5:** Integration of quantitative and qualitative findings

Key themes	Quantitative findings	Qualitative findings	Examples of representative quotations	Interpretation of integration or otherwise
Importance of birth preparedness and complications readiness	60%: Counselled on HF delivery/SBA, 41%: Counselled on saving money or making arrangements for funds, 15%: Counselled on arrangement for transportation, 12%: Counselled on identifying blood donor/donating blood	Shallow context of BP/CR by the women	*“We buy baby things.”* (P3 KSF, 44 years)	Convergent: The discussants understood the concept of BP/CR, but essentially lacked understanding of key components due to gaps in counselling which can result in the superficial grasp of BP/CR exhibited by the women
BP/CR practices in the community	25% of the respondents did not deliver in HF nor had skilled delivery	Fear of caesarean section (C/S), blood donation, and preference for special concoctions and spiritual powers to override the recommendation of surgical delivery	*“Our pregnant women think that Ile Alagbo (TBA) is preferred for delivery if the baby is coming with leg*,* or with hand because the health workers will tell them that they will have C/S but if it is TBA*,* they will do it traditionally to make the baby come out quickly.”* (P1, KSF, 28 years)	Convergent: Some women do not utilize institutional/skilled delivery due to their socio-cultural beliefs and values
	73.4% of the women were not well prepared	Spontaneous emphasis on emergency preparedness was generally lacking in the FGD	*“Some women don’t really prepare. They use Ile Alagbo (TBA) who is living close to their house.”* (P2, IFK, 23 years)	Convergent: Women’s assumption of safe delivery and easy access to TBAs within the community reduces proactive emergency preparedness efforts.
Determinants of BPCR	Lower income/allowance below the *minimum wage (AOR: 1.73, 95% CI: 1.23, 2.42) increased the likelihood of adequate BP/CR practices	Competing socio-cultural financial obligations tied to child delivery such as ‘naming ceremony’	*“You need savings. That is why some people prefer to go to ‘Ile Alagbo’ (TBA) which is cheaper so that they can have money for the naming ceremony*,* to buy things for people during the naming. You really have to prepare yourself financially…….”* (P4, IFK, 40 years)	Divergent: Women with lower economic standing aim to prevent catastrophic health spending that comes with emergencies and hence were better prepared. Qualitative data are subjective but highlight the socio-cultural determinants of BP/CR which are not evident from the quantitative data
	25% of the respondents did not deliver in HF nor had skilled delivery Previous health facility delivery (AOR: 1.58, 95% CI: 1.05, 2.36) increased the likelihood of adequate BP/CR practices	Women save money by subscribing to alternative maternity care providers who allow payments by instalments and long-term debt which is not obtainable in the health facilities.	*“Some of the pregnant woman say that in the hospital*,* the money is too much and so they call home nurse to come and take their delivery. Some after taking their delivery*,* they will not pay immediately*,* some will be paid a little amount. It still boils down to what we are talking about*,* money….”* (P5, KSF, 25 years)	Convergent: Some women opt for cheaper delivery options like TBAs or home births with instalment payments due to financial constraints
		Some women do not like public health facilities because of health system factors like bureaucratic bottlenecks, (for e.g. Attendance is by appointment only) and poor attitude of health workers.	*“Our health centre closes maybe 5 or 6 o’clock and you know some women deliver in the night or in the midnight and they would have closed there*,* it’s not 24 hours* *that is why some women prefer to go elsewhere rather than health centre”.* (P2, KSF, 39 years) *“Some women complain about nurses*,* that they used to insult them….”* (P4, KSF, 40)	Convergent: Inadequacies and gaps in health system responsiveness hinder HF/skilled delivery utilization

*Equivalent to about 90 USD at the time of study; HF=Health facility; SBA=Skilled birth attendant; TBA=Traditional birth attendant

## Discussion

This urban Nigerian study included mostly women in their thirties who were educated, employed, and low-income. Overall BP/CR practices were poor, with only one-quarter classified as ‘well prepared’ (meeting ≥ 3 of 4 key components). Predictors of being well prepared were older age, lower income, lower parity, recent delivery, prior facility delivery, and final BP/CR decisions made by the woman or relatives. FGDs identified barriers: traditional obligations, bureaucratic delays, lack of funds, and poor health worker attitudes.

While women generally understood BP/CR and agreed it was important for maternal and newborn survival, knowledge of specific components was limited. Preparation often focused on baby items, and many acknowledged that few women actually prepare.

In the quantitative study, although only two-fifths of the respondents reported receiving counselling on saving money, most (95%) had saved money or made financial arrangements for childbirth and possible emergencies. This is comparable to the 96.8% from Edo State [30], 92% and 90% reported from other cities in southwestern states of Osun and Ekiti respectively [31], ^,^ [32] but higher than rates from Ogun State and another Lagos study, both (67%) [14, 27]. East African countries reported figures ranging from 54 to 87% [16, 17, 19, 33], while Cameroon was 83% [34]. Financial preparation constitutes an important component of BP/CR and lack of it limits access to adequate care and emergency services thereby increasing the risk of preventable death. In contrast, most discussants in a qualitative study in Ghana did not save money [35]. Poor financial preparation was also reported in other LMIC studies in Ethiopia, Ghana, and India where less than half of respondents reported saving money [18, 21, 23, 35]. Differentials may be related to culture or family dynamics.

Most respondents made no emergency transport arrangements, increasing risk of second-phase delay in reaching care, consistent with findings from neighboring Ghana [21]. This may reflect assumptions of uncomplicated delivery after normal ANC and beliefs that advance planning undermines expectations of a positive outcome. Poor transportation readiness (< 40%) were also documented in East Africa across Ethiopia, Kenya and Tanzania [16, 17, 19, 33]. Confidence in advanced transport networks common in cities could hve also played a role.

Across urban areas in western Nigeria, community-based studies found considerably higher transport preparation rates ranging from 61 to 89% [27, 31, 32]. and 51% from a facility-based study [14]. It was also better in Cameroun (57%) [34], and Farta District in Ethiopia [18]. Beyond the region, a recent systematic review and meta analysis in India with higher economic status than Nigeria equally found a poor rate of 46% [23]. 

Similarly, for the prevention of the most frequent cause of maternal death, excessive bleeding [36, 37], most of our respondents did not adhere to the key BP/CR component of donating blood or identifying in advance, a blood donor(s). It was the least practiced key component among them. The same pattern was observed in several other studies in LMICs in East and West Africa and India [16–19, 22, 23, 27, 30, 33, 34], including facility based studies [14, 38]. In Ekiti and Osun States - both located in southwest Nigeria but less urban than Lagos - figures were higher at 60.8% and 34% respectively, yet this remained the least practiced key component of BP/CR [31, 32]. Blood donation or identification of a potential donor should be a stronger focus of education for expectant women, yet our findings showed that it received the least counselling. This low adherence may reflect limited awareness, misconceptions, or traditional beliefs about blood donation. Across Sub-Saharan Africa, misconceptions and cultural beliefs have likewise been identified as major deterrents to voluntary blood donation [39, 40]. 

Pre-selecting a health facility/SBA for delivery is key to reducing delays in accessing care for normal and complicated deliveries. Most of our respondents fulfilled this BP/CR component, consistent with findings from studies in West Africa, East Africa, and India [17, 18, 23, 27, 30–35]. In other similar studies in East Africa and Ghana, they did not, recording just about one-third compliance (35.8%, 38.5%, and 30.5%) [16, 19, 21]. In Lagos State, some PHCs do not offer delivery services, forcing women to travel farther for care. FGD participants cited this as a challenge, highlighting the need for emergency transport planning. Many women preferred nearby TBA centers to avoid perceived facility demands, unwanted procedures, costs, and poor staff attitudes. Similar findings have been reported in Lagos and Tanzania [41, 42]. Some women also preferred TBAs because of the belief that they possess ‘special powers’ to manage foetal malpresentations, a belief previously documented in Lagos State [41, 43]. Such preferences may increase the risk of maternal and perinatal deaths. FGD participants also cited long distance, long waiting times, and non-24-hour operation as barriers to using public health facilities for delivery. These are key areas for policy and health system intervention.

Overall, preparedness for birth complications was low, with only about one in four respondents ‘well prepared’. Despite most having ANC and institutional delivery histories, BP/CR rates remained low. This observation may be an exposition of potential gaps in service quality, counselling, or health system responsiveness existing in such an urban area like Lagos. Analysis of national data in Ethiopia reported inadequate coverage and substantial gaps in BP/CR counselling messages predicted by spatial differences [44], potentially affecting practice. Many studies including systematic review and meta-analysis across Africa have reported low levels of BP/CR (less than 50%) [16–21], while India, other local (Ogun, Osun, Ekiti, Lagos and Edo States Nigeria) and regional studies (Tanzania) had better overall practice ranging from 50 to 94.4% [14, 23, 27, 30–33]. Variations may result from measurement methods and location. The predictors also vary. Women in our study with lower income were better prepared. This unexpected result could be likened to reports from a similar urban study in Tanzania where having jobs (higher income) negatively affected financial preparation for birth and possible complications [45]. This may reflect underlying social support mechanisms or informal networks warranting further exploration. One could posit that, despite limited spending power, respondents anticipated that inadequate emergency preparedness would incur greater eventual costs, thus motivating them to take necessary precautions. Again, FGDs suggested that men usually provided financial support, thereby compensating for their spouses’ low income. Across SSA, BP/CR was better with increased finances [16, 34, 46], as well as spouse employment [33]. 

Lower parity was a predictor of BP/CR, likely because women with fewer children have more time to attend ANC and are therefore more exposed to BP/CR messaging. They also have greater capacity to make adequate preparations for birth and complications. In contrast, BP/CR was lower among Ethiopian women with higher parity of two or more [16]. Older age as a predictor of BP/CR aligns with studies from Gammo Goffa, Ethiopia, and Ghana [16, 21]. This is likely because older women tend to have more experience and health awareness including familiarity with BP/CR messages. Socially, they usually have stronger support networks and decision-making power in the household. A contrast was seen in Sodo town, another part of southern Ethiopia [17], where younger age predicted BP/CR. Yoruba ethnicity predicted BP/CR. As Yoruba is the predominant language in the study area, and is commonly used for health messages in facilities, non-Yoruba speakers may miss key BP/CR information. Women or their relatives making the final BP/CR decision also significantly increased the likelihood of being ‘well prepared’, given the risk to mother and newborn in emergencies.

More recent birth and facility delivery also predicted BP/CR. This may reflect better recall of the pregnancy, recent health system improvements, and increased BP/CR counselling in facilities. Similarly, health facility delivery was also a predictor in Kenya and Tanzania [19]. 

Counselling on facility delivery/SBA predicted adequate BP/CR, likely by raising awareness. Similar findings were reported in East African countries of Ethiopia [16, 17], and Somaliland [47]. 

FGDs identified lack of finance as a key barrier to BP/CR due to competing demands. Many women prioritize funds for the traditional Yoruba ‘naming ceremony’ on the 8th day after birth, which can be costly. This financial pressure drives some to choose home delivery or TBAs, perceived as cheaper than facility birth. This qualitative finding contrasts with quantitative data, suggesting women may reserve money for the ceremony rather than for obstetric emergencies. Ghanaian discussants equally mentioned finances as the major challenge to BP/CR and most did not set aside funds [35]. Qualitative methods are interpretive and context-specific but remain highly valuable for understanding lived experiences, context, and meaning that may not be fully captured through quantitative approaches.

Although Lagos State’s Community-based Health Insurance Scheme (CBHIS) covers maternity care, insured women showed no significantly better BP/CR practices, suggesting coverage alone is insufficient [48, 49]. While recent West African evidence links health insurance to improved maternal and child service access, equitable and sustainable implementation remains needed [50]. Strengthening financial protection for maternal health is therefore critical to reducing preventable maternal and neonatal mortality.

Other documented predictors of women being ‘well prepared’ for obstetric complications in Africa which were not reflected in our study include: better knowledge of danger signs [33], having ANC or higher number of ANC contacts [14, 16, 18, 19, 33, 34, 46, 51], higher educational level [17–21, 33, 51], history of still birth [18, 51], and lower spousal age difference [32]. 

FGD findings indicated that men were the main financial drivers of BP/CR, suggesting some level of involvement. Although decisions were reportedly made jointly, many women may have deferred to men’s choices because of limited financial empowerment. This underscores the need for multi-pronged BP/CR interventions that promote women’s economic empowerment while encouraging male involvement beyond financial support to other BP/CR components. Over the years, the importance of male partner involvement in reproductive, maternal, newborn and child health (RMNCH) has been emphasized and promoted for better outcomes [52, 53]. In Sub-Saharan Africa, low levels of partner involvement reduces BP/CR, further highlighting the importance [46]. 

There are other strategies that can address the multidimensional nature of BP/CR challenges. Community-based programmes and conditional cash transfers (CCTs) could be implemented to promote equity. These have shown promising results in Nigeria and Ethiopia [20, 54–56]. 

### Strengths and limitations

This study adds to the body of knowledge on the elimination of preventable maternal and neonatal deaths in urban areas in a low resource setting. It was community-based involving a large sample size and had a qualitative component for more insight and context. In addition, we concentrated on the key components for measurement of BP/CR. These attributes increase the validity of results. However, there are some limitations. Due to the cross-sectional nature, causative inferences cannot be made. Inferential analysis depended predominantly on demographic and socioeconomic variables than primary effects which may create a risk of interpreting model outputs to cultural characteristics. In addition, such models may produce overly optimistic predictions for vulnerable members of groups that show overall positive outcomes at the population level. Generalizability of findings beyond urban settings is limited, only two FGDs were done, and there is also possibility of recall bias which was minimized by including only women with recent births (2 years).

### Implications of findings

Health education and campaigns on BP/CR key components should be implemented. Community-based culturally sensitive education may additionally help de-emphasize expensive traditional social obligations surrounding childbirth, such as elaborate naming ceremonies, which were identified during FGDs as contributing to financial strain and poor preparedness. Such education could also be utilized to promote male involvement, concepts of health insurance and CCTs. Collaboration with local commercial drivers through their trade union may be explored to facilitate emergency transportation plans. This could be a viable option in a city like Lagos. Through policy reforms and training, the health system barriers such as extending facility operating hours and poor health worker attitudes could be addressed.

## Conclusion

Our findings highlight critical gaps in BP/CR, increasing the risk for maternal and perinatal deaths in this urban setting. We urge integrated interventions addressing individual, community, and health system barriers. These include strengthen standardized BP/CR counselling during ANC, engage trained maternal health volunteers to support community mobilization of women and men to improve BP/CR knowledge, as previously demonstrated in urban settings [57], engage traditional gatekeepers and community leaders in prioritizing culturally sensitive messaging, and reform facility-level barriers.

Future directions include the design, implementation and scaling of effective BP/CR interventions alongside longitudinal studies to evaluate their impact, and addressing blood donation gaps. These efforts can accelerate progress toward reducing maternal and neonatal deaths and achieving SDG 3.

## Data Availability

The datasets used and/or analyzed during the study are available from the corresponding author on reasonable request.

## Declarations

The research was conducted in accordance with the Declaration of Helsinki.

## Abbreviations

ANC: Antenatal care
AOR: Adjusted Odds Ratio
BP/CR: Birth preparedness and complications readiness
CCTs: Conditional cash transfers
CI: Confidence Interval
COR: Crude Odds Ratio
C/S: Caesarean section
EmOC: Emergency obstetric care
FGD: Focus Group Discussion
Health facility: HF
HW: Health worker
IFK: Ifako–Ijaiye
JHPIEGO: Johns Hopkins Program for International Education in Gynaecology and Obstetrics
KSF: Kosofe
LGA: Local Government Area
LMIC: Low–and middle–income countries
MDGs: Millennium Development Goals
MNCH: Maternal, Newborn and Child Health
MMR: Maternal Mortality Ratio
NMR: Neonatal Mortality Rate
RA: Research assistant
RMNCH: Reproductive, maternal, newborn and child healthcare
SD: Standard deviation
SDGs: Sustainable Development Goals
TBA: Traditional Birth Attendant
USD: United States Dollar
